# The moderating role of absorptive capacity and the differential effects of acquisitions and alliances on Big Pharma firms' innovation performance

**DOI:** 10.1371/journal.pone.0172488

**Published:** 2017-02-23

**Authors:** K. D. S. Fernald, H. P. G. Pennings, J. F. van den Bosch, H. R. Commandeur, E. Claassen

**Affiliations:** 1 Erasmus School of Economics, Applied Economics Department, Erasmus University, Rotterdam, Netherlands; 2 VU University, Biology and Society Research Department (Athena Institute), Faculty of Earth and Life Sciences, Amsterdam, Netherlands; Utrecht University, NETHERLANDS

## Abstract

In the context of increased pharmaceutical innovation deficits and Big Pharma blockbusters’ patent expirations, this paper examines the moderating role of firms’ absorptive capacity in external innovation activities of Big Pharma firms. The study indicates a rising interest of Big Pharma in acquisitions of and alliances with biotechnology companies. Unfortunately, this increased interest is not reflected in the number of new drugs generated by Big Pharma. We find that acquisitions of biotech companies have negatively affected Big Pharma firms’ innovation performance on average but these acquisitions might have a positive effect at higher levels of acquiring firms’ absorptive capacity. Moreover, also acquisitions of pharma companies and alliances with biotech companies only have a positive effect on innovation performance at sufficiently high levels of absorptive capacity. The moderating role of absorptive capacity implicates that a tight integration of internal R&D efforts and (unrelated) external knowledge is crucial for harnessing complementarity effects.

## Introduction

The pharmaceutical industry is one of the most research-intensive industries, with average new product development (NPD) trajectories of 11.9 years [1, 2]. For the past decade the industry has been coping with a growing “productivity gap” [3, 4] or “productivity paradox” [5], which is generally described as a decrease in new products launched versus an increase in research and development (R&D) expenditures [6]. These increased expenses appear to be related to an increasingly rigid regulatory environment and higher quality demands [7, 8]. In addition, patent expiry on numerous blockbuster drugs, also referred to as the “patent cliff”, and consequent generic competition is currently affecting the industry, eroding $billions in annual sales [9]. Although the decrease in pharmaceutical productivity is controversial [8], the combination of challenges that are involved has serious ramifications for maintaining the industry’s margins and double-digit growth rates of past decades [10]. These rates are still incorporated into the growth expectations of shareholders and can be maintained only with an annual launch of at least two new ‘blockbuster’ drugs [5].

**Fig 1:**
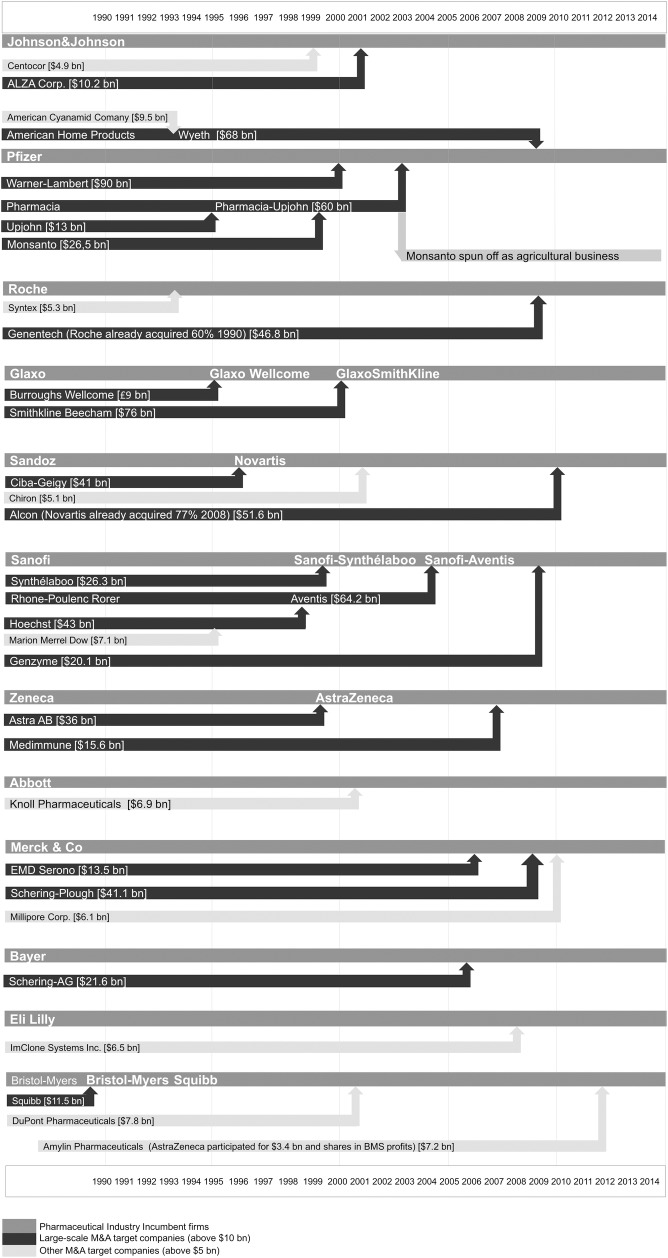
Big Pharma consolidation. M&As that led to the current 12 largest Big Pharma firms. (source: SDC Platinum Database (ThomsonReuters))

An important strategy that has been used over the past two decades to address thinning pipelines involves mergers and acquisitions (M&As) [11, 12]. Fig 1 shows the consolidation of Big Pharma, with 32 incumbent firms in 1990 merging to 12 firms in 2013.

**Fig 2:**
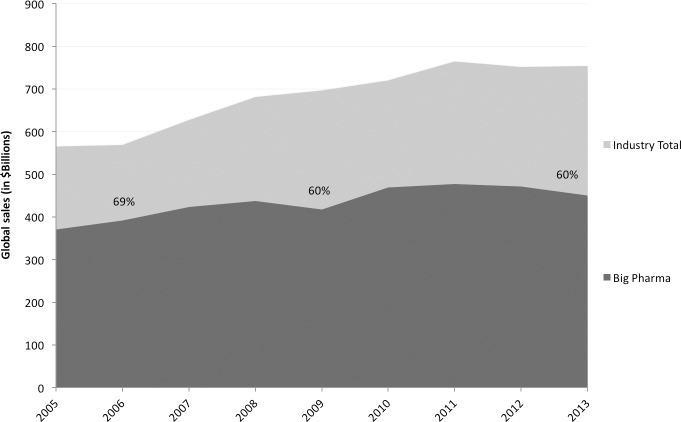
Global pharmaceutical sales (in $Billions). Showing the proportion of global sales of the Big Pharma firms as illustrated in Fig 1 compared to global industry sales. (source: EvaluatePharma; Datastream(ThomsonReuters))

Together, these firms have generated more than 60% of the combined global pharmaceutical sales over the past decade (see Fig 2). Therefore, in this study, these 12 firms are considered to represent Big Pharma, which is the nickname given to the most influential global pharmaceutical firms. According to Danzon et al. [13] and Frantz [14], M&As appear to be a response to the expected patent expirations and gaps in a firm’s product pipeline. However, this consolidation strategy has had little effect, as M&As do not appear to create or destroy value [11]. Nonetheless, since the 1980s, pharmaceutical firms have employed this M&A strategy with respect to biotechnology small- and medium-sized enterprises (SMEs) in the hopes of countering innovation deficits [15, 16].

The current innovation challenges have coincided with the considerable rise of new sources of innovation for pharmaceutical firms. The biotech ‘revolution’, which began in the 1970s [17], has significantly affected the radical innovation process within the industry. This rise of scientific drug discovery, spurred the origination of highly innovative and specialized biotechnology-driven firms [18].

**Fig 3:**
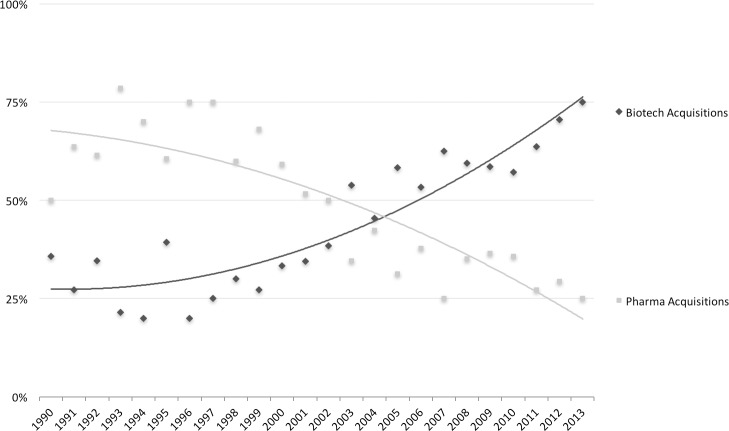
Trends in externally acquired knowledge and assets through acquisitions by big pharma firms between 1990 and 2013. Showing the acquisitions of ‘Pharma’ targets and ‘Biotech’ targets as a percentage of included acquisitions. (source: SDC Platinum Database (Thomson & Reuters))

**Fig 4:**
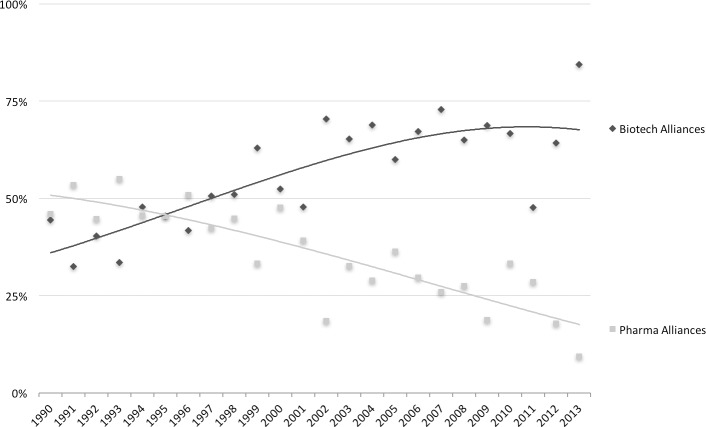
Trends in externally acquired knowledge and assets through alliances of big pharma firms between 1990 and 2013. Showing access to knowledge/assets in alliances with ‘Pharma’ companies and ‘Biotech’ companies as a percentage of all studied alliances.(source: SDC Platinum Database (Thomson & Reuters))

Biotechnology as a new source of external innovation was expected to be the answer to the challenges that the pharmaceutical industry is currently confronted with [19]. This development resulted in a continuous trend in the formation of new pharma-biotech collaborations and acquisitions of biotech SMEs [20, 21] (Figs 3 and 4). However, the coincidence of the incumbent firms’ focus on external sources of innovation, the considerable rise in R&D expenditures, and the stagnant pattern of newly developed drugs (i.e. productivity paradox), does raise questions regarding the effects of external innovation from biotechnology SMEs on incumbent pharmaceutical firms’ innovation performance.

The (bio)pharmaceutical industry is a prime example where technologically unrelated innovation sources are used to replenish incumbent firms’ R&D pipelines, making it ideal to investigate differential effects of related and unrelated acquisitions and alliances on firms’ innovation performance. By establishing a framework based on the innovation activities of incumbent pharmaceutical firms, this paper uniquely aims to explore effects of related and unrelated sources of innovation, accessed through alliances and acquisitions, on firms’ innovation performance. Moreover, the aim is to show the moderating role of absorptive capacity, measured as internal R&D, in these effects, identifying either complementarity or substitutability between Big Pharma firms’ internal and external R&D activities.

The paper is organized as follows. Section 2 provides the theoretical background and derives several hypotheses. In section 3, we discuss the data and methodology of our study. Section 4 presents the results, and section 5 concludes the paper.

## Theory and hypotheses

This study applies the resource-based view (RBV) [22] on external innovation from a firm-level perspective, where M&As and alliances are modes to acquire or access non-substitutable and non-imitable resources to enhance firms’ innovation performance. From this theoretical perspective previous research shows that M&As in the pharmaceutical and biotechnology industry are positive for both parties only when the acquirer and the target own and combine strategically valuable resources and capabilities [23]. Regarding alliances, resource alignment seems to be crucial as well [24].Furthermore, in the context of open innovation [25] and exploration and exploitation [26] as it applies to the (bio)pharmaceutical industry [27, 28], three important innovation activities are internal R&D efforts, engaging in alliances, and engaging in M&As. Finally, this theory draws upon real option theory, which predicts that firms learn through an alliance and will acquire its alliance partner when the alliance turns out to be a success [29–32].

In the pharmaceutical industry, several types of M&As are prevalent, which include but are not limited to: large-scale mergers between pharmaceutical firms (e.g., Glaxo Wellcome merging with Smithkline Beecham to form GlaxoSmithkline in 2000 and Sanofi merging with Aventis in 2004), large-scale biotech mergers (e.g., Biogen and Idec merging in 2003) and acquisitions of biotech SMEs [20] on the one hand, and acquisitions of pharmaceutical companies on the other hand. We argue that pharma companies are more related to Big Pharma firms, in terms of business logic, knowledge, technologies and NPD; than biotech companies. Therefore, in this paper, biotech companies are considered relatively unrelated to the incumbent pharma firms.

Incumbent (pharma) firms and biotech companies make alliance and acquisition deals to reach their respective goals. Biotech companies want to ensure their short-term survival by accessing financial resources, sharing the high risks associated with drug development and ultimately gaining market access. In contrast, pharmaceutical firms intend to acquire product candidates with blockbuster potential to fill gaps in their pipeline [33, 34]. Although the blockbuster model is being increasingly questioned by firms themselves [5], the strong focus on the short-term commercial exploitation of high-potential products appears to resonate in the strategies of firms. As a result, firms generally target profitable or later clinical stage biotech companies. Although early stage companies are less expensive to acquire, they are typically more distant from becoming profitable and thus are less appealing [35]. The targets also tend to be acquired for only one or a few products from their pipeline [36], resulting in a frequent write-off of acquired in-process R&D.

### External innovation activities and innovation performance

Alliances generally seem to outperform acquisitions when it comes to effects on a firm’s innovation performance [37]. Where overall effects of alliances on innovation are often positive [38–41], the effects of acquisitions on innovation are mostly neutral or negative [37]. Effects of acquisitions on various performance measures appear to be more negative when acquirers and targets have more diverging knowledge bases or are more dissimilar in size [42]. So, especially for unrelated acquisitions, where the assimilation and application of newly acquired knowledge are likely to be resource consuming and can be counter-productive [43], the effects are found to be negative [44–47]. In many cases acquisitions also fail due to other reasons such as resistance to change and the not invented here (NIH) syndrome [48].

Compared to acquisitions, different dynamics play a role in alliances, and hence different management capabilities are needed to adequately exploit innovation accessed through strategic alliances [49]. There are various types of strategic alliances, in this paper we include ‘joint ventures’ (JVs), ‘in-license deals’, ‘funding of external R&D projects’, and ‘collaborative R&D agreements’ as alliances, if they were considered to be directly relevant for NPD. Alliances are often engaged prior to M&As [50], presenting the opportunity of ‘cherry-picking’ at a relatively low cost before committing to all assets of a target company [37, 51]. However, the extent to which alliances have positive effects on innovation performance is highly dependent on various factors, such as the relatedness of the knowledge bases of involved firms, the intensity of collaboration, and optimal alliance networks [37, 46]. Especially relatedness may be an important factor and positive effects of alliances may be greater for related alliances as opposed to unrelated alliances. Distinguishing between biotech and pharma alliances, Deeds and Hill [41], however, find no significant differences in innovation performance between both types of alliances.

### The moderating role of absorptive capacity

Absorptive capacity is originally defined by Cohen and Levinthal [52] as a firm-level construct regarding a firm’s ability ‘to recognize the value of new, external knowledge, assimilate it, and apply it to commercial ends’ (p. 128). They suggested that absorptive capacity is largely a function of the firm’s level of prior related knowledge, and that it is critical to the firm’s innovative capabilities. In various studies, a firm’s internal R&D investments is considered to be a proxy for its absorptive capacity [52–60] and appears to be a contingency variable that critically influences the relationship between external R&D strategies and innovation performance [61–63]. In particular R&D acquisitions are complementary innovation activities at higher levels of internal R&D investments (i.e. higher expenditures on internal R&D, absolute or relative to sales), while at lower levels, internal R&D and acquisitions turn out to be substitutive strategic options. This might especially be true for related acquisitions as literature typically suggests that creating economies of scale and scope require a high level of technology- and market-relatedness [37, 64]. Accordingly, Zahra and Hayton [53] find positive significant interaction effects of related acquisitions and absorptive capacity on both firms’ ROE (return on equity) and revenue growth. Building upon these arguments, we derive the following hypothesis:

Hypothesis 1a: Absorptive capacity has a positive moderating effect on the relationship between pharma acquisitions and Big Pharma firms’ innovation performance.

Pisano [65] explained that the rapid internalization of biotechnological R&D through acquisitions is likely to be an undesirable model. He argued that acquiring biotech SMEs can be particularly dangerous when used to overcome internal deficits. Acquiring biotechnology companies is only recommended after sufficient accumulation of in-house R&D experience [65–67]. Correspondingly, Miyazaki [68] has reported negative effects in high-tech industries when firms choose between either high levels of internal R&D (i.e., ‘making’) or external growth strategies involving M&As (i.e., ‘buying’).

Although, as H1a suggests, complementarity between internal and external R&D often depends on relatedness [37, 64, 69], positive, but mostly non-significant, interaction effects of internal R&D activity and unrelated knowledge acquisitions have been found as well [53, 70]. In such cases, innovation management requires a tight integration of internal and external knowledge to capture the positive effects each innovation activity has on the marginal return of the other [69]. It seems that increased absorptive capacity (i.e. internal R&D) could positively moderate the effects of unrelated acquisitions on firms’ innovation performance. As such, the following hypothesis is suggested:

Hypothesis 1b: Absorptive capacity has a positive moderating effect on the relationship between biotech acquisitions and Big Pharma firms’ innovation performance.

A very fundamental difference between alliances and acquisitions lies in the degree of ownership between the parties involved. While larger firms can play a dominant role, the ownership of external R&D remains with the other firm, resulting in a lack of ownership advantages that could be essential in creating complementarity between internal and related external R&D. The choice between internal R&D and related alliances is influenced by whether they are complements or substitutes which, ultimately, rely on whether synergies exist between them [71] and the R&D governance mode choice appears to be an important contingent variable in this regard [72]. Due to a higher degree of separation in terms of ownership and governance between internal R&D and external alliances, firms are more likely to choose M&As over alliances with increased relatedness [72]. For alliances, there seems to be an optimum as neither too much nor too little relatedness contributes to firm innovation [73]. Related alliances and internal R&D may be substitutable, so that the marginal benefit of pharma alliances could decrease with higher levels of internal R&D investments. This would lead to a negative moderating effect of absorptive capacity (substitution). Whereas Zahra and Hayton [53] find a positive moderating role of absorptive capacity on the financial effects of related alliances, Berchicci [74] also finds a substitution effect between a firm’s internal R&D capacity and external R&D through licensing, alliances and technology agreements with other firms. Given these findings, a substitution effect between internal R&D and related external innovation through alliances may be expected in the context of this study as well. The following hypothesis is formulated.

Hypothesis 2a: Absorptive capacity has a negative moderating effect on the relationship between pharma alliances and Big Pharma firms’ innovation performance.

The effects of unrelated alliances on firms’ innovation performance, on the other hand, could be enhanced by firms’ absorptive capacity. Laursen [75] explains that the inherent tensions and conflicts between exploratory and exploitative activities may call for organizational separation of these activities within firms. Perhaps a higher degree of separation in governance between the (more explorative) activities of biotechnology firms and the (explorative and exploitative) activities of pharmaceutical firms can result in increased complementarity effects on innovation when internal pharmaceutical R&D interacts with biotech R&D through strategic alliances. Correspondingly, Lavie et al. [76] explain how inter-organizational R&D alliances may involve varying degrees of basic research and incremental development in which they recognize intermediate activities that combine new knowledge development and the leveraging of prior knowledge. Increased internal explorative R&D might enhance effects of partnerships with unrelated innovation on a firm’s innovation performance. This corresponds with positive and significant empirical findings from existing literature [53, 54, 70], offsetting possible negative effects from opportunism in unrelated alliances. Considering this, the following hypothesis is suggested:

Hypothesis 2b: Absorptive capacity has a positive moderating effect in the relationship between biotech alliances and Big Pharma firms’ innovation performance.

Alliances may be created as a means to learn about the effectiveness of a partner prior to an acquisition. Real options theory predicts that the option to acquire the alliance partner will only be executed when circumstances are favorable [29–32]. In our case, favorable outcomes can be interpreted as success in terms of innovation. When the outcome of the alliance is unfavorable (yet), the alliance will be continued or dissolved. As a result, an acquisition that follows an alliance provides the acquirer with useful experience and such an acquisition is expected to have a positive effect on innovation performance. Hence, we propose the following hypothesis.

Hypothesis 3: Acquisitions that follow an alliance have a positive effect on Big Pharma firms’ innovation performance.

## Methodology

In this study, we collect data related to all incumbent pharmaceutical firms, together known as Big Pharma. As shown in Fig 1, these firms are: Johnson & Johnson, Pfizer, Roche, GlaxoSmithKline, Novartis, Sanofi-Aventis, AstraZeneca, Abbott, Merck & Co (and Schering), Bayer, Eli Lilly, and Bristol-Myers Squibb. We examine the effects of alliances and acquisitions that occurred between the period from January 1990 to December 2013.

### Measures

#### Innovation performance

Abundant previous studies have used both input measures (e.g., R&D expenditures) and output measures (e.g., product introductions) to study innovation performance. According to De Man and Duysters [37], output measures are generally preferred over input measures and are also expected to provide the most accurate measure of innovation performance, particularly when estimating the effects of M&As. Accordingly, an output measure was used for the analysis in this study.

The Center for Drug Evaluation and Research (CDER) distinguishes eight different chemical classes of new drug approvals (NDAs), of which the first class concerns new molecular entities (NMEs); defined as drugs that contain an active moiety that has never been approved by the FDA or marketed in the US. This class has been used in the literature as a measure for pharmaceutical innovation performance [11, 77–80]. In addition, products generated by biotechnologies such as recombinant DNA technology are registered as Biologic License Applications (BLAs), governed by the Center for Biologics Evaluation and Research (CBER).

The total number of NMEs and BLAs generated by the Big Pharma firms and their subsidiaries during the studied period, as depicted in Fig 1, was used as the dependent variable in this study. The data was obtained from the online databases of the FDA, CDER, and CBER.

NMEs and BLAs generated by firms prior to their merger were included in the analysis. NMEs and BLAs generated by subsidiaries that remained active were only included after the respective acquisition, and only if the subsidiary also engaged in alliances and acquisitions prevalent in our dataset. Subsequently, we excluded NMEs and BLAs with the same name, applicant and approval date as a previous one. These occur due to multiple registrations according to different dosages or delivery methods. A total number of 318 NMEs and BLAs were included in the analysis (S1 File).

The focus was on US approvals because the USA is the largest market for pharmaceuticals and biotech products and accounts for more than 50% of global pharmaceutical sales [81] and any firm that generates new drugs would take advantage of this market.

#### Acquisitions

From ThomsonReuters SDC Platinum M&A database, an initial total number of 1,205 mergers and acquisitions was gathered for the 12 firms (Fig 1). This dataset was analyzed per entry in order to properly categorize and include or exclude individual acquisitions. As discussed earlier, this study does not focus on large-scale M&As, as depicted in Fig 1 (transaction values over $10 billion); therefore, these were excluded from the analysis, and all minority stake acquisitions were also excluded. For mergers of firms that both acquired smaller companies during the studied period, acquisitions were added together. Subsequently, it was determined whether acquisitions were directly relevant for NPD, possibly leading to either an NME or a BLA. On the basis of this premise, acquisitions of (research) services companies and medical devices/diagnostic product companies were excluded from the analysis.

The remaining acquisition targets were subsequently categorized either as being biotechnology companies or pharmaceutical companies. This categorization was mainly based on the respective target company’s lead product(s) in development. Information from the deal synopsis as included in the database was used in combination with additional searches for company websites and profiles on websites (e.g. Bloomberg’s businessweek.com). The criteria used for distinguishing pharma companies from biotech companies resemble those described in existing literature [82–85]. Targets were considered to be pharmaceutical companies if their products (in development) mainly concerned small molecule drugs and/or the companies used more traditional drug discovery methods for lead generation. For biotech targets, consistent with literature [18, 82], we identified different types in our dataset. We only included Chiesa and Chiarioni’s [82] description of “core biotech companies”, which include “product biotechs”, “drug agent biotechs”, and “platform biotechs”. Targets were considered to be biotech companies if their products (in development) concerned products of biotechnologies (e.g. recombinant proteins, antibodies) or platform biotechnologies (relating to gene therapy or cell therapy, for example).

After these exclusion steps a total of 568 acquisitions were included in the analysis (S2 File), of which 290 acquisitions of pharmaceutical companies (i.e. *Pharma Acquisitions*) and 278 acquisitions of biotechnology companies (i.e. *Biotech Acquisitions*).

#### Alliances

With the first inquiry, a total of 2,878 alliances were extracted from ThomsonReuters SDC Platinum alliances and joint venture database. This dataset was again analyzed per entry, using similar exclusion criteria as described above. Again, alliances of firms that merged during the studied period were added together. First, the type of alliance was considered and ‘alliances for services’, ‘out-licensing’, ‘marketing alliances’, ‘out-sourcing’, and ‘manufacturing alliances’ were excluded from the analysis. As a consequence, only ‘joint ventures’ (JVs), ‘in-license deals’, ‘funding of external R&D projects’, and ‘collaborative R&D agreements’ were included, if these were considered to be directly relevant for NPD (i.e. marketing or manufacturing JVs were also excluded). In addition, these latter set of alliance types with (research) services and medical devices/diagnostic product partners were also excluded from the analysis, as such partners were again not considered to be directly relevant for NPD.

The remaining alliances were further categorized based on the alliance partner being either a biotechnology partner or a pharmaceutical partner. For this categorization the same criteria as described for the acquisition targets, were used. Similarly, this categorization was also based on the deal synopsis as provided by the SDC Platinum database or, if necessary, on additional online data.

After these exclusion steps a total of 1,270 alliances were included in the analysis (S3 File), of which 552 alliances with pharmaceutical companies (i.e. *Pharma Alliances*) and 718 alliances with biotechnology companies (i.e. *Biotech Alliances*).

#### Absorptive Capacity (ACAP)

As stated by Zahra and Hayton [53], several measures for absorptive capacity have been used in the literature, but the most popular measure is R&D spending, as a firm’s internal R&D is the foundation of its absorptive capacity [52]. Similarly to Lin et al. [54], we used a relative measure for R&D intensity, which was generated by dividing the R&D expenditures by the sales of the respective big pharma firms as shown in Fig 1.

Both data on firms’ R&D expenditures and sales was collected from ThomsonReuters’ Datastream and these measures were added together for merged firms prior to their merger. The role of *Absorptive Capacity* was assessed by estimating effects of interactions with the acquisitions and alliances variables on firms’ innovation performance. For the main effects this measure for R&D intensity was included as a control variable in models without firm dummies.

*Size–*Firms’ size was included as a control variable, measured by the number of employees. This data was also obtained from Datastream. In the analysis a log transformation of this variable was generated.

#### Models and analysis

For the regression analyses, a panel dataset was used to estimate effects on the dependent variable, *Innovation Performance*_*it*_ for firm *i* in year *t*. Independent variables were generated by creating stock variables for the measures as described above for firm *i* over the period from year *t* to *t-5*, hereby creating a time lag of 5 years (similar effects were found using lags of 4 and 6 years). This time lag was introduced to estimate the lagged effects of our independent variables, considering companies’ stage in product development. A time lag of 5 years seems to be sufficient [86], and a longer time lag would lead to a larger decrease in the number of observations.

First, the main effects were estimated with a Poisson regression with robust standard error (SE) corrections, with and without firm fixed effects, including *Absorptive Capacity*_*i(t—t-5)*_ and *ln(Size)*_*i(t—t-5)*_ as control variables.

Subsequently, the moderating role of absorptive capacity was explored with a Poisson regression of the same models including interactions of absorptive capacity with the acquisitions and alliances variables, using the same time lags of 5 years. By using dummy variables, we control for time-specific and firm-specific effects. The hypotheses were tested using the following model:

$$\begin{array}{l} Innovation\;Performance_{it}=\\ \alpha +\beta_{1}ACAP_{i\left(t-t-5\right)}+\beta_{2}ln{\left(SIZE\right)}_{i\left(t-t-5\right)}+\beta_{3}Pharma\;Acquistions_{i\left(t-t-5\right)}+\\ \beta_{4}Biotech\;Acquisitions_{i\left(t-t-5\right)}+\beta_{5}Pharma\;Alliances_{i\left(t-t-5\right)}+\\ \beta_{6}Biotech\;Alliances_{i\left(t-t-5\right)}+\beta_{7}ACAP*Pharma\;Acquisitions_{i\left(t-t-5\right)}+\beta_{8}ACAP*\\ Biotech\;Acquisitions_{i\left(t-t-5\right)}+\beta_{9}ACAP*Pharma\;Alliances_{i\left(t-t-5\right)}+\\ \beta_{10}ACAP*Biotech\;Alliances_{i\left(t-t-5\right)}+\varepsilon_{it} \end{array}$$

## Results

### Acquisitions and alliances

Over the studied period of 24 years (1990–2013), we do not observe a rise in the total number of acquisitions. However, pharmaceutical acquirers seem to have shifted their focus, with respect to the type of acquisition targets they acquire. Fig 3 shows an increase in *Biotech Acquisitions* and a decline in *Pharma Acquisitions*, as percentages of total acquisitions that were considered to be directly relevant for NPD. This trend supports the notion of increased investments in biotech by Big Pharma firms.

Regarding the total number of alliances, we observe a decline, decreasing from an annual average of 220 between 1990 and 1995 to an annual average of 55 between 2008 and 2013. Similar to the patterns regarding acquisitions, the results show that over the past 24 years, firms have developed an increasing preference for collaborations in which they gain access to biotech products and platform technologies (Fig 4). This result is not surprising, as many acquisitions, especially small-scale acquisitions, are preceded by alliances and collaborations [50]. These trends regarding acquisitions and alliances correspond to the trends that have been reported in other studies [20, 21].

### Innovation performance

**Fig 5:**
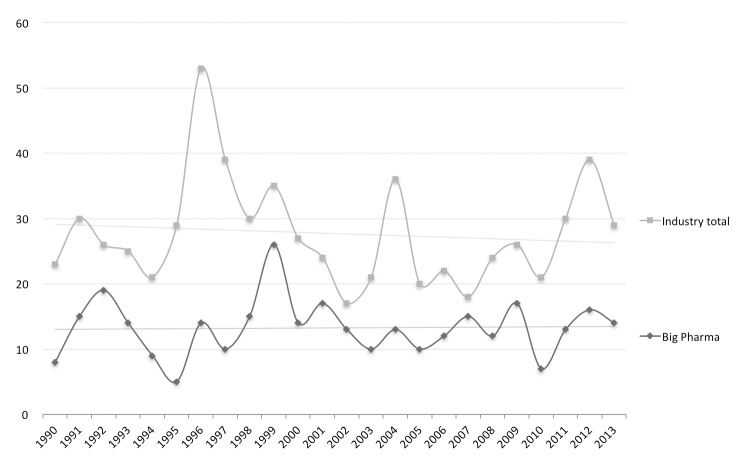
Output in terms of NMEs and BLAs produced by the Big Pharma versus the industry as a whole. These results represent the output NMEs and BLAs from the 12 largest pharma firms and the output of the industry as a whole based on all drugs approved by the FDA. (source: CDER (Center for Drug Evaluation and Research) and FDA (Food and Drug Administration))

The total number of NMEs and BLAs generated by Big Pharma was used as the measure for innovation performance of these firms. In correspondence with existing literature [8, 17, 19], these measures show a rather static pattern for both Big Pharma and the entire industry (Fig 5).

Based on these numbers, Big Pharma accounts for close to 50% of all NME approvals over the studied period of 24 years (1990–2013). Considering the vast increases in R&D spending, these results are considered representative for the productivity gap as described in literature [5, 6, 87, 88].

### Main effects

The descriptive statistics for all variables are provided in Table 1. We observe an expected positive correlation between the control variable, *Size*, and the dependent variable, *Innovation Performance*.

In addition, *Size* correlates with all acquisitions and alliances variables, which is not surprising as larger firms would also be able to engage in more acquisitions and alliances. Interestingly, there is no significant correlation between *Absorptive Capacity* (i.e. R&D intensity) and *Size* or *Innovation Performance*. And, *Innovation Performance* only correlates with *Acquisitions* and *Pharma Acquisitions*, in addition to *Size*, while *Absorptive Capacity* only correlates with *Acquisitions*, *Biotech Acquisitions* and *Pharma Alliances*.

**Table 1:** Descriptive statistics.

	*Mean*	*Std*. *Dev*.	*Min*.	*Max*.	*1*	*2*	*3*	*4*	*5*	*6*	*7*	*8*
*1*. *Innovation performance*	1.1	1.22	0	6								
*2*. *Size* ^*a*^	11.33	.558	10.1	12.6	.222*							
*3*. *Absorptive Capacity*	.139	.058	.052	.422	.084	.072						
*4*. *Acquisitions*	2.13	2.07	0	9	.190*	.440*	.157*					
*5*. *Pharma Acquisitions*	1.01	1.34	0	6	.179*	.397*	-.015	.751*				
*6*. *Biotech Acquisitions*	.965	1.21	0	7	.095	.275*	.267*	.740*	.165*			
*7*. *Alliances*	4.77	4.61	0	30	.077	.168*	-.137	.169*	.196*	.049		
*8*. *Pharma Alliances*	1.92	2.51	0	17	.064	.170*	-.181*	.133*	.230*	-.030	.875*	
*9*. *Biotech Alliances*	2.49	2.4	0	15	.077	.154*	-.038	.185*	.120*	.132*	.826*	.492*

*Correlation is significant at the 5% level (2-tailed).

^a^ Log transformation

Table 2 displays the main effects without the use of firm dummies. Here, we show that the control variable *Size* is positive and significant in our models. In addition, Table 2 shows that *Absorptive Capacity* (i.e. R&D intensity) has a positive but non-significant effect on the innovation performance of Big Pharma firms. This provides empirical support for the previously described innovation paradox, where significant increases in R&D expenditures have not lead to increases in firms’ innovation performance [5, 6].

**Table 2:** Main effects on Big Pharma firm's innovation performance (without firm dummies).

Dependent Variable: *Innovation Performance*	Model 1		Model 2	
*Size*	.353**	[.134]	.310*	[.138]
*Absorptive Capacity*	.776	[1.05]	.809	[1.09]
*Acquisitions*	.021	[.011]	-	
*Pharma Acquisitions*	-		.021	[.015]
*Biotech Acquisitions*	-		.024	[.020]
*Alliances*	.008*	[.004]	-	
*Pharma Alliances*	-		.015	[.012]
*Biotech Alliances*	-		.009	[.012]
*Constant*	-5.15**	[1.76]	-4.73**	[1.81]
*Adjusted R-squared*	.12		.12	
*N*	228		228	
*Log pseudolikelihood*	-414.97		-412.67	

Year dummies are included, and most are not significant (not shown).

* Statistically significant at the 5% level.

** Statistically significant at the 1% level.

Table 3 displays the main effects estimated with the Poisson regression analysis for the same models, including firm dummies (A negative binomial regression analysis provided similar results; not shown). Overall, acquisitions negatively affect the innovation performance of Big Pharma firms. This effect can be primarily attributed to the negative effect of *Biotech Acquisitions* as opposed to *Pharma Acquisitions*. These findings are consistent with the literature. *Pharma Acquisitions* also appear to be negative but are non-significant, which supports the notion that technologically related acquisitions are more beneficial for a firm’s innovation performance than unrelated acquisitions.

**Table 3:** Main effects on Big Pharma firm's innovation performance.

Dependent Variable: *Innovation Performance*	Model 1		Model 2	
*Size*	-.594*	[.255]	-.694**	[.258]
*Absorptive Capacity*	-3.14	[2.09]	-3.55	[2.17]
*Acquisitions*	-.051**	[.016]	-	
*Pharma Acquisitions*	-		-.039	[.022]
*Biotech Acquisitions*	-		-.043*	[.019]
*Alliances*	.011**	[.004]	-	
*Pharma Alliances*	-		.029***	[.008]
*Biotech Alliances*	-		-.008	[.010]
*Constant*	7.76*	[3.35]	8.97**	[3.41]
*Adjusted R-squared*	.23		.24	
*N*	228		228	
*Log pseudolikelihood*	-358.78		-358.25	

Year dummies and firm dummies are included, and most are not significant (not shown).

* Statistically significant at the 5% level.

** Statistically significant at the 1% level.

*** Statistically significant at the .1% level.

In contrast to acquisitions and as expected, main effects of alliances positively affect innovation performance, primarily because of the positive and significant effect of *Pharma Alliances*, again illustrating the benefits of relatedness. There appears to be a negative but non-significant relationship between *Biotech alliances* and *Innovation performance*. Nevertheless, alliances with biotech partners outperform acquisitions of these companies and may, therefore, be a more preferred strategy, in particular when considering the moderating effects of firms’ absorptive capacity.

### Interaction effects

The moderating role of *Absorptive capacity* is shown in Table 4. Although the interaction effect with acquisitions is not significant, it is positive, while the main effect is negative and significant. *Absorptive capacity* seems to predominantly moderate the effects of related acquisitions, given that the interaction with *Pharma acquisitions* is positive and significant, which provides empirical evidence for hypothesis 1a. On the other hand, the interaction with *Biotech acquisitions* is positive but not significant, suggesting that absorptive capacity does play a moderating role here, neutralizing the negative main effect of biotech acquisitions. However, this effect is not significant, providing insufficient support for hypothesis 1b.

**Table 4:** Interaction effects on Big Pharma firm's innovation performance.

Dependent Variable: *Innovation Performance*	Model 3		Model 4		Expected Sign
*Size*	-.650**	[.252]	-.576*	[.261]	
*Absorptive Capacity*	-2.34	[3.85]	-8.51*	[3.46]	
*Acquisitions*	-.060*	[.030]	-		
*Pharma Acquisitions*	-		-.089*	[.039]	
*Biotech Acquisitions*	-		-.080**	[.030]	
*Alliances*	.026***	[.008]	-		
*Pharma Alliances*	-		.110***	[.022]	
*Biotech Alliances*	-		-.052*	[.022]	
ACAP * Acquisitions	.143	[.170]	-		
ACAP * Pharma Acquisitions	-		.568**	[.220]	H1a (+)
ACAP * Biotech Acquisitions	-		.274	[.173]	H1b (+)
ACAP * Alliances	-.152*	[.063]	-		
ACAP * Pharma Alliances	-		-.758***	[.177]	H2a (-)
ACAP * Biotech Alliances	-		.405*	[.172]	H2b (+)
*Constant*	9,10**	[3.20]	8.70**	[3.35]	
*Adjusted R-squared*	.28		.29		
*N*	228		228		
*Log pseudolikelihood*	-386.64		-381.67		

Year dummies and firm dummies are included, and most are not significant (not shown).

* Statistically significant at the 5% level.

** Statistically significant at the 1% level.

*** Statistically significant at the .1% level.

For alliances, overall, *Absorptive capacity* seems to negatively moderate their positive main effect. However, this seems to be caused by a stronger negative and significant interaction effect of *Absorptive capacity* and *Pharma alliances*, while the interaction of *Absorptive capacity* and *Biotech alliances* is positive and significant. These results support hypotheses 2a and 2b.

### Alliances as real options

Considering hypothesis 3, Table 5 shows regression results where a distinction was made between acquisitions with experience (acquisitions where there was a previous alliance between the acquirer and the target) and acquisitions without experience. Approximately 21% of all the acquired companies in the dataset engaged in an alliance with the acquirer prior to the acquisition. For the combined measure of innovation performance (NMEs and BLAs) as dependent variable we find a significantly negative effect of acquisitions without partner specific experience, but we did not find a positive effect for partner specific experience. However, after examining NMEs and BLAs as separate dependent variables, we find that partner specific experience tends to have a positive, but non-significant, effect on innovation performance when considering biologics (BLAs), which include most biotechnological products (e.g. recombinant proteins, antibodies, recombinant vaccines). Thus, experience through alliance prior to acquisitions seems to benefit the effects of acquisitions on the acquirers’ innovation performance more when it concerns biologics output. Though the positive effect that is found for acquisitions with experience in the case of BLAs is not significantly different from zero, it is significantly different (at the 5% level) from the effect found for acquisitions without experience. For NMEs, the negative effect of acquisitions on innovative performance persists, regardless of whether the acquisition was preceded by an alliance or not. Overall, we do not find support for hypothesis 3.

**Table 5:** Main effects of acquisitions with partner specific experience.

Dependent Variable:	*NMEs and BLAs*		*NMEs*		*BLAs*		Expected Sign
*Size*	-.780	[.460]	-.855	[.446]	-.413	[.887]	
*Absorptive Capacity*	-2.51	[3.20]	-3.62	[3.17]	4.82	[8.25]	
*Acquisitions with experience*	-.050	[.046]	-.065	[.048]	.028	[.104]	H3 (+)
*Acquisitions without experience*	-.041*	[.020]	-.033	[.021]	-.066*	[.033]	
*Alliances*	.010	[.007]	.013	[.008]	.006	[.012]	
*Constant*	10.56	[6.05]	11.55	[5.89]	-12.73	[11.25]	
*Adjusted R-squared*	.14		.10		.29		
*N*	228		228		228		
*Log pseudolikelihood*	-335.09		-294.65		-120.27		

Year dummies and firm dummies are included, and most are not significant (not shown).

* Statistically significant at the 5% level.

## Conclusions and discussion

In this study we show that acquisitions of biotech companies have an overall negative effect on Big Pharma firms’ innovation performance. However, the level of these firms’ absorptive capacity, which is characterized by a relative measure for in-house R&D investments, is a contingency variable that critically influences the relationship between some external innovation activities and Big Pharma firms’ innovation performance. In particular, acquisitions of both pharma and biotech companies are complementary innovation activities at higher levels of absorptive capacity (threshold at ACAP > 0.16 (p < .000) for pharma acquisitions and at ACAP > 0.29 (p < .005) for biotech acquisitions), whereas the general effects of these acquisitions appear to be negative. Noteworthy, pharma acquisitions outperform biotech acquisitions in this regard, illustrating the known influence of technology- and market-relatedness. In addition, we show that the same complementarity exists between biotech alliances and absorptive capacity, while pharma alliances’ main effect is positive but these alliances turn out to be substitutive strategic options at higher levels of absorptive capacity. Experience with acquisition targets through a previous alliance may also significantly weaken the negative effect on innovation performance.

Given the current innovation deficits Big Pharma is confronted with, this study indicates that Big Pharma firms may have neglected internal R&D efforts because of the promising expectations of the biotech revolution. Apparently, firms have relied on biotech companies for innovation and may have underestimated the need for an emphasis on internal R&D, absorptive capacity, and post-acquisition integration. We show that optimal gain from external technologically unrelated innovation, either through acquisitions or alliances, is contingent upon these closely related constructs; and when underemphasized, effects of engaging with external biotech innovation can be detrimental to a pharmaceutical firm’s innovation performance.

Although literature mostly attributes the M&A strategy of incumbent firms to their drying pipelines and need for innovation, the negative main effects of biotech acquisitions could also be explained by differences in acquisition motives. Schweizer [33] indicates that M&A strategies certainly differ and that an understanding of the motives behind them is important for the successful implementation of different types of acquisitions. Moreover, Ahuja and Katila [47] acknowledge that technological reasons do not motivate all acquisitions. Other motives may include the desire to obtain access to distribution channels, to gain entry into new markets, or to obtain financial synergies or market power [47]. Furthermore, Ahuja and Katila [47] argue that such acquisitions cannot be expected to improve an acquiring firm’s innovation performance. Moreover, R&D replenishment in the short-term or other short-term motives, such as the ability to enter new biotechnology markets or enhance short-term competitive advantages, could be driving the spur of acquisitions of biotechnology companies, which could not reasonably be expected to resort real benefits to innovation performance.

Where general effects of acquisitions on innovation performance are mainly negative, effects of alliances are mainly positive. Alliances with either related or unrelated partners may be subject to differences in tensions between, for example, vigilance and trust, control and autonomy, design and emergence, innovation and replication, and exploration and exploitation, referring to dialectical theory [89–91]. Such tensions can strengthen an alliance and increase its likelihood of success; however, they can also lead to alliance instability and consequent negative effects. These tensions could thus influence internal R&D processes and, consequently, the interaction effects with internal R&D intensity on a firm’s performance. In this regard, technology relatedness seems to be an important factor as higher levels of absorptive capacity (i.e. R&D intensity) are complementary with biotech alliances but substitutable with pharma alliances.

A related explanation for the negative interaction effect of alliances with pharma companies is that a large number of such partners could lead to more interference and reconciliation issues regarding strategy and thus internal R&D processes. Governance mode choice in inter-firm cooperation [72, 92] may be important here as well as, in contrast, interaction effects of pharma acquisitions and absorptive capacity are positive. We argue that this difference may be attributed to potential ownership advantages of acquirers during acquisitions and post-acquisition integration, where the acquirer exerts a more dictating role, fitting the acquired assets into its own strategy and R&D focus.

### Implications

For future pharmaceutical productivity and business model innovation [93], it is essential that acquired biotechnology companies, once integrated, form a complementary force with internal pharmaceutical R&D efforts, even when companies are acquired for reasons other than R&D replenishment and NPD. Other researchers have concluded that focusing either on accumulating internal R&D but not exploring external opportunities or on continuously acquiring but not assimilating new knowledge will negatively affect innovative performance [68, 94]. A delicate balance must be found between the ‘make’ and ‘buy’ strategies [68], and ideally a focus on both is most beneficial as capabilities associated and developed by putting effort in the ‘make’ increase absorptive capacity necessary to optimally leverage the ‘buy’ [67]. In addition to a balance between ‘make’ and ‘buy’, a similar balance between ‘make’ and ‘collaborate’ is equally important and may vary for different types of partners and targets. Thus, a tight integration of externally acquired knowledge and internal R&D efforts is crucial for harnessing potential complementarity effects [69]. This encompasses an important firm level implication regarding R&D management, emphasizing the importance of this integration, especially with respect to technologically unrelated companies. For affairs with related companies, firms are best to focus on gaining governance control over external R&D through acquisitions as this can work complementary to internal R&D efforts. Without such control, this cooperation with external related companies will be substitutable with internal R&D efforts.

Fetterhoff and Voelkel [95] describe the management of open innovation activities in the context of biotechnology by proposing a five-stage value chain: 1) ‘seeking’ opportunities, 2) ‘evaluating’ the market potential of an opportunity, 3) ‘recruiting’ potential partners, 4) ‘capturing’ value through rapid commercialization, and 5) ‘extending’ the innovation (i.e., working collaboratively to generate additional innovation and develop collaboration beyond the life cycle of a given product). From the firm perspective, this five-stage process represents a process of integrating external explorative innovation, adequately exploiting that innovation, and eventually further ‘extending’ the innovation in an explorative manner. Absorption and diffusion of innovation are dependent on the organizational structure in question [96]. Each stage offers an opportunity for value creation but also presents unique challenges requiring specific capabilities. Examining the results of this study, we suggest that large pharmaceutical firms often do not possess such specific capabilities. Moreover, pharmaceutical firms might not complete this value chain through the final step of ‘extending’ externally acquired innovation. Generating additional innovation beyond the life cycle of one or a few products requires exploratory capabilities and increases the value of the initially acquired innovation [95].

The results in this study do not imply that firms should make fewer investments in biotechnologies. On the contrary, we believe that investment in and adequate exploitation of biotechnologies holds the future for pharmaceutical productivity, innovation and growth. However, we suggest that it is unwise to fully rely on acquiring biotechnology innovation alone, while neglecting to continuously invest in internal explorative R&D activities [67], needed for increased absorptive capacity and post-acquisition integration capabilities. Investing in biotechnology requires a long-term perspective that includes future internal exploration and that will not be successful if the post-acquisition integration process is predominantly focused on short-term innovation boosts and short-term profits. The motives behind acquisitions and alliances are important, as they may function as a predictor of the success of an acquisition or alliance and of whether such activity will positively affect innovation performance.

### Limitations and further research

Our results should be interpreted with caution in view of the limitations of this study. Although the trends that are identified in our study are consistent with global industry trends, this study was conducted using data of the largest pharmaceutical firms of the past decades, which makes it difficult to generalize these results and conclusions throughout the industry, including smaller (bio)pharmaceutical firms. However, the purpose of this study was to investigate the processes pertaining to large incumbent pharmaceutical firms. Additional data related to smaller firms would have increased the amount of data but could have also obscured the effects that are mostly associated to Big Pharma firms’ conduct of business. Furthermore, our dataset is quite substantial, as the Big Pharma firms account for more than 60% of global pharmaceutical sales over the past decade (see Fig 2) and have produced close to 50% of all approved NMEs and BLAs between 1990 and 2013.

Another limitation of this study is the extent to which we defined our categories. Perhaps additional categorization would reveal more nuances; for example, different therapeutic areas or types of products may be associated with different effects on innovation. In addition, in this study we used but one variable that could moderate general effects of acquiring innovation, while additional variables could also play an important role herein (e.g. measures for alliance or acquisition experience).

Noteworthy, this study was limited to analyzing a specific industry with a high-risk profile that is increasingly dependent on innovation from a still upcoming industry. As such, similar effects might be apparent in other industries with similar characteristics. Further research could examine such industries in a similar way to assess this. Another avenue of further research could be to study innovation performance from the perspective of the acquisition target, in this case the biotechnology company (for example see: Fernald et al. [92]), as most related research has focused on the incumbent firm’s perspective. Additional further research could include assessing, in detail, the determinants of the absorptive capacity of firms, and the necessary capabilities of optimal post-acquisition integration.

Finding a direct relation between innovative input and trends in output remains difficult. However, by implementing time lags of up to five years in the models, we have been able to measure significant differences in the effects of acquisitions and alliances on innovation. As data from before 1990 was not included, this study does not allow for an accurate measurement of long-term effects beyond five years. However, we show that acquiring biotechnology companies will not solve the innovation deficit in the next five years without continuous development of internal R&D.

## Supporting information

S1 File Launched drugs of big pharma firms between 1990 and 2013. This file includes the collected data on new drugs (NDAs, NMEs and BLAs) from big pharma firms as analyzed for this study.
(XLSX)

S2 File Acquisitions of big pharma firms between 1990 and 2013. This file includes the collected data on acquisitions by big pharma firms as analyzed for this study.
(XLSX)

S3 File Alliances of big pharma firms between 1990 and 2013. This file includes the collected data on alliances by big pharma firms as analyzed for this study.
(XLSX)

## References

[1] DiMasi JA, Hansen RW, Grabowski HG. The price of innovation: new estimates of drug development costs. Journal of health economics. 2003;22(2):151–85. 10.1016/S0167-6296(02)00126-1 12606142

[2] Pronker E, Weenen T, Commandeur H, Osterhaus A, Claassen H. The gold industry standard for risk and cost of drug and vaccine development revisited. Vaccine. 2011;29(35):5846–9. 10.1016/j.vaccine.2011.06.051 21722688

[3] Ashburn TT, Thor KB. Drug repositioning: identifying and developing new uses for existing drugs. Nature reviews Drug discovery. 2004;3(8):673–83. 10.1038/nrd1468 15286734

[4] Pronker ES, Weenen TC, Commandeur H, Claassen EH, Osterhaus AD. Risk in vaccine research and development quantified. PloS one. 2013;8(3):e57755 10.1371/journal.pone.0057755 23526951 PMC3603987

[5] Gassmann O, Reepmeyer G. Organizing pharmaceutical innovation: from science‐based knowledge creators to drug‐oriented knowledge brokers. Creativity and Innovation Management. 2005;14(3):233–45.

[6] Fernald KDS, Weenen TC, Sibley KJ, Claassen E. Limits of biotechnological innovation. Technology and Investment. 2013;4:168–78.

[7] Cohen FJ. Macro trends in pharmaceutical innovation. Nature Reviews Drug Discovery. 2005;4(1):78–84. 10.1038/nrd1610 15688075

[8] Schmid EF, Smith DA. Keynote review: Is declining innovation in the pharmaceutical industry a myth? Drug discovery today. 2005;10(15):1031–9. 10.1016/S1359-6446(05)03524-5 16055019

[9] Kessel M. The problems with today's pharmaceutical business [mdash] an outsider's view. Nature biotechnology. 2011;29(1):27–33. 10.1038/nbt.1748 21221096

[10] Dhankhar A, Evers M, Møller M. Escaping the sword of Damocles: toward a new future for pharmaceutical R&D. McKinsey Perspect Drug Device R&D. 2012.

[11] Munos B. Lessons from 60 years of pharmaceutical innovation. Nature Reviews Drug Discovery. 2009;8(12):959–68. 10.1038/nrd2961 19949401

[12] De Ruiter J, Holston PL. Drug patent expirations and the “patent cliff”. US Pharm. 2012;37(6):12–20.

[13] Danzon PM, Epstein A, Nicholson S. Mergers and acquisitions in the pharmaceutical and biotech industries. Managerial and Decision Economics. 2007;28(4‐5):307–28.

[14] Frantz S. Pipeline problems are increasing the urge to merge. Nature Reviews Drug Discovery. 2006;5(12):977–9. 10.1038/nrd2206 17201023

[15] Drews J. Innovation deficit revisited: reflections on the productivity of pharmaceutical R&D. Drug Discovery Today. 1998;3(11):491–4.

[16] Drews J, Ryser S. Innovation deficit in the pharmaceutical industry. Drug Information Journal. 1996;30(1):97–108.

[17] Hopkins MM, Martin PA, Nightingale P, Kraft A, Mahdi S. The myth of the biotech revolution: An assessment of technological, clinical and organisational change. Research policy. 2007;36(4):566–89.

[18] Chiaroni D, Chiesa V, Frattini F. Patterns of collaboration along the bio-pharmaceutical innovation process. Journal of Business Chemistry. 2008;5(1):6.

[19] Nightingale P, Martin P. The myth of the biotech revolution. TRENDS in Biotechnology. 2004;22(11):564–9. 10.1016/j.tibtech.2004.09.010 15491800

[20] Mittra J. Life science innovation and the restructuring of the pharmaceutical industry: Merger, acquisition and strategic alliance behaviour of large firms. Technology Analysis & Strategic Management. 2007;19(3):279–301.

[21] Roijakkers N, Hagedoorn J. Inter-firm R&D partnering in pharmaceutical biotechnology since 1975: Trends, patterns, and networks. Research Policy. 2006;35(3):431–46.

[22] Barney J. Firm resources and sustained competitive advantage. Journal of management. 1991;17(1):99–120.

[23] Grill P, Bresser RK. Resource-based theory and mergers & acquisitions success. 2011.

[24] Das TK, Teng B-S. A resource-based theory of strategic alliances. Journal of management. 2000;26(1):31–61.

[25] Chesbrough HW. Open innovation: The new imperative for creating and profiting from technology: Harvard Business Press; 2003.

[26] March JG. Exploration and exploitation in organizational learning. Organization science. 1991;2(1):71–87.

[27] Andriopoulos C, Lewis MW. Exploitation-exploration tensions and organizational ambidexterity: Managing paradoxes of innovation. Organization Science. 2009;20(4):696–717.

[28] Gilsing V, Nooteboom B. Exploration and exploitation in innovation systems: The case of pharmaceutical biotechnology. Research Policy. 2006;35(1):1–23.

[29] Warner AG, Fairbank JF, Steensma HK. Managing uncertainty in a formal standards-based industry: A real options perspective on acquisition timing. Journal of Management. 2006;32(2):279–98.

[30] Kogut B, Kulatilaka N. Operating flexibility, global manufacturing, and the option value of a multinational network. Management science. 1994;40(1):123–39.

[31] Leiblein MJ. The choice of organizational governance form and performance: Predictions from transaction cost, resource-based, and real options theories. Journal of management. 2003;29(6):937–61.

[32] Reuer JJ, Tong TW. Real options in international joint ventures. Journal of Management. 2005;31(3):403–23.

[33] Schweizer L. Organizational integration of acquired biotechnology companies into pharmaceutical companies: The need for a hybrid approach. Academy of Management Journal. 2005;48(6):1051–74.

[34] Amir-Aslani A, Negassi S. Is technology integration the solution to biotechnology's low research and development productivity? Technovation. 2006;26(5):573–82.

[35] Willyard C. Profit-hungry pharma sees some biotechs as ripe for the picking. Nature medicine. 2009;15(5):466–. 10.1038/nm0509-466b 19424184

[36] Rydzewski RM. Real world drug discovery: A chemist's guide to biotech and pharmaceutical research: Elsevier; 2010.

[37] De Man A-P, Duysters G. Collaboration and innovation: a review of the effects of mergers, acquisitions and alliances on innovation. Technovation. 2005;25(12):1377–87.

[38] Inoue H, Liu Y-Y. Revealing the intricate effect of collaboration on innovation. PloS one. 2015;10(3):e0121973 10.1371/journal.pone.0121973 25799138 PMC4370822

[39] Danzon PM, Nicholson S, Pereira NS. Productivity in pharmaceutical–biotechnology R&D: the role of experience and alliances. Journal of health economics. 2005;24(2):317–39. 10.1016/j.jhealeco.2004.09.006 15721048

[40] Arora A, Gambardella A, Magazzini L, Pammolli F. A breath of fresh air? Firm type, scale, scope, and selection effects in drug development. Management Science. 2009;55(10):1638–53.

[41] Deeds DL, Hill CW. Strategic alliances and the rate of new product development: an empirical study of entrepreneurial biotechnology firms. Journal of Business Venturing. 1996;11(1):41–55.

[42] Hagedoorn J, Duysters G. The effect of mergers and acquisitions on the technological performance of companies in a high-tech environment. Technology Analysis & Strategic Management. 2002;14(1):67–85.

[43] Haspeslagh PC, Jemison DB. Managing acquisitions: Creating value through corporate renewal: Free Press New York; 1991.

[44] Hitt MA, Hoskisson RE, Ireland RD, Harrison JS. Effects of acquisitions on R&D inputs and outputs. Academy of Management Journal. 1991;34(3):693–706.

[45] Keil T, Maula M, Schildt H, Zahra SA. The effect of governance modes and relatedness of external business development activities on innovative performance. Strategic Management Journal. 2008;29(8):895–907.

[46] Cloodt M, Hagedoorn J, Van Kranenburg H. Mergers and acquisitions: Their effect on the innovative performance of companies in high-tech industries. Research policy. 2006;35(5):642–54.

[47] Ahuja G, Katila R. Technological acquisitions and the innovation performance of acquiring firms: A longitudinal study. Strategic management journal. 2001;22(3):197–220.

[48] Katz R, Allen TJ. Investigating the Not Invented Here (NIH) syndrome: A look at the performance, tenure, and communication patterns of 50 R & D Project Groups. R&D Management. 1982;12(1):7–20.

[49] Dyer JH, Singh H. The relational view: Cooperative strategy and sources of interorganizational competitive advantage. Academy of management review. 1998;23(4):660–79.

[50] Al-Laham A, Schweizer L, Amburgey TL. Dating before marriage? Analyzing the influence of pre-acquisition experience and target familiarity on acquisition success in the “M&A as R&D” type of acquisition. Scandinavian Journal of Management. 2010;26(1):25–37.

[51] Duysters G, Man AP. Transitory alliances: an instrument for surviving turbulent industries? R&D Management. 2003;33(1):49–58.

[52] Cohen WM, Levinthal DA. Absorptive capacity: a new perspective on learning and innovation. Administrative science quarterly. 1990:128–52.

[53] Zahra SA, Hayton JC. The effect of international venturing on firm performance: The moderating influence of absorptive capacity. Journal of Business Venturing. 2008;23(2):195–220.

[54] Lin C, Wu Y-J, Chang C, Wang W, Lee C-Y. The alliance innovation performance of R&D alliances—the absorptive capacity perspective. Technovation. 2012;32(5):282–92.

[55] Belderbos R, Carree M, Diederen B, Lokshin B, Veugelers R. Heterogeneity in R&D cooperation strategies. International journal of industrial organization. 2004;22(8):1237–63.

[56] Meeus MT, Oerlemans LA, Hage J. Patterns of interactive learning in a high-tech region. Organization Studies. 2001;22(1):145–72.

[57] Oltra MJ, Flor M. The impact of technological opportunities and innovative capabilities on firms' output innovation. Creativity and Innovation Management. 2003;12:137–44.

[58] Stock GN, Greis NP, Fischer WA. Absorptive capacity and new product development. The Journal of High Technology Management Research. 2001;12(1):77–91.

[59] Tsai W. Knowledge transfer in intraorganizational networks: Effects of network position and absorptive capacity on business unit innovation and performance. Academy of management journal. 2001;44(5):996–1004.

[60] Flatten TC, Engelen A, Zahra SA, Brettel M. A measure of absorptive capacity: Scale development and validation. European Management Journal. 2011;29(2):98–116.

[61] Hagedoorn J, Wang N. Is there complementarity or substitutability between internal and external R&D strategies? Research Policy. 2012;41(6):1072–83.

[62] Catozzella A, Vivarelli M. The catalysing role of in-house R&D in fostering complementarity among innovative inputs. Industry and Innovation. 2014;21(3):179–96.

[63] Tsai K-H, Wang J-C. External technology acquisition and firm performance: A longitudinal study. Journal of Business Venturing. 2008;23(1):91–112.

[64] Cassiman B, Colombo MG, Garrone P, Veugelers R. The impact of M&A on the R&D process: An empirical analysis of the role of technological-and market-relatedness. Research Policy. 2005;34(2):195–220.

[65] Pisano GP. The governance of innovation: vertical integration and collaborative arrangements in the biotechnology industry. Research Policy. 1991;20(3):237–49.

[66] Grimpe C. Successful product development after firm acquisitions: The role of research and development. Journal of Product Innovation Management. 2007;24(6):614–28.

[67] Hoang H, Rothaermel FT. Leveraging internal and external experience: exploration, exploitation, and R&D project performance. Strategic Management Journal. 2010;31(7):734–58.

[68] Miyazaki H. An analysis of the relation between R&D and M&A in high-tech industries. Applied Economics Letters. 2009;16(2):199–201.

[69] Cassiman B, Veugelers R. In search of complementarity in innovation strategy: Internal R&D and external knowledge acquisition. Management science. 2006;52(1):68–82.

[70] Rothaermel FT, Hess AM. Building dynamic capabilities: Innovation driven by individual-, firm-, and network-level effects. Organization Science. 2007;18(6):898–921.

[71] Ceccagnoli M, Higgins M, Palermo V. Internal R&D versus in-licensing complements or substitutes.

[72] Riccobono F, Bruccoleri M, Perrone G. External knowledge sourcing for R&D activities: antecedents and implications of governance mode choice. Technology Analysis & Strategic Management. 2014;(ahead-of-print):1–19.

[73] Sampson RC. R&D alliances and firm performance: The impact of technological diversity and alliance organization on innovation. Academy of Management Journal. 2007;50(2):364–86.

[74] Berchicci L. Towards an open R&D system: internal R&D investment, external knowledge acquisition and innovative performance. Research Policy. 2013;42(1):117–27.

[75] Laursen K. Keep searching and you’ll find: what do we know about variety creation through firms’ search activities for innovation? Industrial and Corporate Change. 2012;21(5):1181–220.

[76] Lavie D, Stettner U, Tushman ML. Exploration and exploitation within and across organizations. The Academy of Management Annals. 2010;4(1):109–55.

[77] Graves SB, Langowitz NS. Innovative productivity and returns to scale in the pharmaceutical industry. Strategic Management Journal. 1993;14(8):593–605.

[78] DiMasi JA. New drug innovation and pharmaceutical industry structure: trends in the output of pharmaceutical firms. Drug Information Journal. 2000;34(4):1169–94.

[79] Cardinal LB. Technological innovation in the pharmaceutical industry: The use of organizational control in managing research and development. Organization Science. 2001;12(1):19–36.

[80] Grabowski HG, Wang YR. The quantity and quality of worldwide new drug introductions, 1982–2003. Health Affairs. 2006;25(2):452–60. 10.1377/hlthaff.25.2.452 16522586

[81] EvaluatePharma. Pharmaceutical & Biotech Sales Analysis by Country: Top Drugs, Top Regions. 2014.

[82] Chiesa V, Chiaroni D. Industrial Clusters in Biotechnology: Driving Forces, Development Processes, and Management Practices: Imperial College Press; 2005.

[83] Rothaermel FT, Deeds DL. Exploration and exploitation alliances in biotechnology: a system of new product development. Strategic management journal. 2004;25(3):201–21.

[84] Gottinger H-W, Umali CL. The evolution of the pharmaceutical-biotechnology industry. Business History. 2008;50(5):583–601.

[85] Drakeman DL. Benchmarking biotech and pharmaceutical product development. Nature biotechnology. 2014;32(7):621–5. 10.1038/nbt.2947 25004226

[86] Jeon J, Hong S, Ohm J, Yang T. Causal Relationships among Technology Acquisition, Absorptive Capacity, and Innovation Performance: Evidence from the Pharmaceutical Industry. PloS one. 2015;10(7):e0131642 10.1371/journal.pone.0131642 26181440 PMC4504511

[87] TAIT J, MITTRA J. Industry challenges. Chemistry and industry. 2004;(23):24–5.

[88] Martinez B, Goldstein J. Big pharma faces grim prognosis. Wall Street Journal A. 2007;1:12.

[89] Van de Ven AH, Poole MS. Explaining development and change in organizations. Academy of management review. 1995;20(3):510–40.

[90] Das TK, Teng B-S. Instabilities of strategic alliances: An internal tensions perspective. Organization science. 2000;11(1):77–101.

[91] De Rond M, Bouchikhi H. On the dialectics of strategic alliances. Organization Science. 2004;15(1):56–69.

[92] Fernald KDS, Pennings HPG, Claassen E. Biotechnology Commercialization Strategies: Risk and Return in Interfirm Cooperation. Journal of Product Innovation Management. 2015;32(6)(6):971–96.

[93] Denicolai S, Ramirez M, Tidd J. Creating and capturing value from external knowledge: the moderating role of knowledge intensity. R&D Management. 2014;44(3):248–64.

[94] Cefis E. The impact of M&A on technology sourcing strategies. Economics of Innovation and New Technology. 2010;19(1):27–51.

[95] Fetterhoff TJ, Voelkel D. Managing open innovation in biotechnology. Research-Technology Management. 2006;49(3):14–8.

[96] Sáenz-Royo C, Gracia-Lázaro C, Moreno Y. The Role of the Organization Structure in the Diffusion of Innovations. PloS one. 2015;10(5):e0126076 10.1371/journal.pone.0126076 25978360 PMC4433189

